# It Happened to a Friend of Mine: The Influence of Perspective-taking on the Acknowledgment of Sexual Assault Following Ambiguous Sexual Encounters

**DOI:** 10.1177/0886260520957678

**Published:** 2020-09-29

**Authors:** Veronica M. Lamarche, Laurie James-Hawkins

**Affiliations:** 1 University of Essex, Colchester, Essex, UK

**Keywords:** sexual assault, perspective taking, ambiguity

## Abstract

Failure to acknowledge that one has been the victim of sexual violence is an important, yet understudied, barrier that prevents women from seeking appropriate support following sexual violence. Drawing from a literature of demonstrating the benefits of self-distancing when evaluating emotionally charged personal information, the effects of self-distancing on acknowledgment of sexual assault were tested. Four experimental studies (*N*_total_ = 1,609) manipulated perspective-taking, either by asking women to imagine a series of hypothetical sexual encounters as experiences that happened to themselves or to their friends, or by asking women to describe a sexual experience from a first- or third-person perspective. Findings from the studies suggest that taking another person’s perspective can help women to label ambiguous sexual experiences as more inappropriate and coercive. Notably, this did not seem to stem from women downplaying or dismissing experiences when they imagined themselves, as they reported anticipating more negative and less positive emotions in the scenarios where they imagined themselves compared to a friend. Nonetheless, in spite of the stronger anticipated negative emotional response when imagining themselves, women were less open to information about resources associated with sexual assault and support when they imagined themselves compared to a friend. This pattern of findings replicated for own, past sexual experiences but only to the extent that women spontaneously engaged in distanced perspective-taking themselves. This research suggests in addition to using contextual information to disambiguate and determine whether a sexual experience was inappropriate, taking a distanced perspective might provide a route through which women can come to terms with the experience and open up to the use of community-based services and sexual assault resources.

Sexual violence remains a prevalent social and public health problem worldwide. Global estimates suggest that approximately 35% of women have experienced physical or sexual violence in their lifetimes (World Health Organization, 2013). In the United States, the United Kingdom and Canada alone, estimates suggest that between 25% and 44% of women and 6% and 17% of men have been victims of sexual violence (Office for National Statistics, 2018; Smith et al., 2017; Statistics Canada, 2015). The estimated prevalence rates are worse still in marginalized communities, especially among sexual and racial minorities and among people with disabilities (Benoit et al., 2015). Furthermore, sexual victimization can lead to repeated traumas, with 60% of victims reporting subsequent revictimization (Classen et al., 2005). Sexual violence is therefore a critical issue that cuts across gender, race, religion, and socioeconomic status (McInturff, 2013). Prevailing sexual assault scripts and narratives suggest that it should be easy to identify and label experiences as an assault (Deming et al., 2013). However, in reality, social interactions are often more ambiguous and nuanced which makes it difficult to reconcile whether or not something untoward transpired during a sexual encounter. This lack of clarity over the boundaries that encompass sexual assault add to the difficulty of acknowledging one’s own victimization. Thus, ambiguity presents a unique barrier to acknowledging an act of sexual violence has been committed and accessing the support and resources people need. The current research aims to better understand some of the social and psychological barriers that prevent people from acknowledging the perpetration of sexual violence and from seeking support following assaults. The current research tested whether a perspective-taking intervention could be used to increase acknowledgment of inappropriate sexual behaviors (i.e., coercion, lack of consent, sexual assault, rape) following an ambiguous sexual encounter, and whether the presence of other contextual cues which contribute to ambiguity (e.g., alcohol use, memory loss, evidence of distress) influenced the efficacy of this manipulation.

## Unacknowledged Sexual Assault

There are several well-understood barriers that prevent women from reporting that they have been the victim of sexual assault and seeking help. These include fears of reprisal, stigma surrounding sexual assault, and personal blame (Khan et al., 2018). However, a less understood aspect of unreported sexual assault is a phenomenon known as unacknowledged sexual assault. Unacknowledged sexual assaults are sexual encounters that meet all of the hallmarks of a sexual assault (i.e., coercion, use of force, lack of consent), but which the potential victim claims was not assault (Orchowski et al., 2013; Peterson & Muehlenhard, 2011; Wilson & Miller, 2016). It is believed that approximately 30% to 88% of all sexual assaults remain unacknowledged by the victim, suggesting they are relatively prevalent (Peterson & Muehlenhard, 2011). Despite not acknowledging an assault happened, these experiences impart serious consequences. Victims of unacknowledged sexual assaults exhibit the same negative psychological and physical consequences as sexual assault victims who do acknowledge their assaults (e.g., heightened anxiety; depression) (Wilson et al., 2018). Furthermore, these problems persist longer than for victims who acknowledge their assaults, often because they do not seek out appropriate support services (e.g., community resources; counseling; physicians) (Wilson et al., 2018). This suggests that failing to acknowledge a sexual assault is not an effective strategy for coping or recovering from the experience. Given the serious consequences associated with unacknowledged sexual assaults, psychological interventions that can be leveraged to help people come to terms with their experiences must be explored.

## Ambiguity in Social Interactions

Acknowledging sexual assaults is made more difficult by the ambiguity surrounding many social interactions, including sexual encounters. However, the majority of research examining barriers to acknowledging sexual assault, have focused on interactions that follow traditional rape or sexual assault scripts relying on distress after the encounter, overt use of force or coercion, or unwitting attacks from a violent stranger (Deming et al., 2013). Communicating consent during an intimate encounter can be complicated (Muehlenhard et al., 2016). In an ideal world, people would be able to clearly and unambiguously communicate their wants, needs, and desires. In reality, people rely on nonverbal signals, particularly in sexual contexts, to communicate (Hall, 1998; Hall et al., 2019; Hickman & Muehlenhard, 1999; Muehlenhard et al., 2016).

When interactions are ambiguous, people typically rely on external cues to draw inferences and make attributions for one another’s behaviors (Hall et al., 2019). For example, people might refer to flirtations earlier in the evening as justification for why they continued to be sexually intimate with someone who gave mixed or ambiguous signals later (Metts & Spitzberg, 1996), or strategically use silence to prioritize their own desires, assuming that their partner will verbally interject if they disagreed with the behaviors (Dalessandro et al,, 2019; Hickman & Muehlenhard, 1999). Ambiguity also imbues perceptions and behaviors after the sexual act has occurred. For example, people anticipate that victims will be highly distressed, when in reality they might use humor as a way of coping with the trauma (Besser et al., 2015; Martin et al., 1993) or simply show no evidence of distress when recounting their experience (Klippenstine & Schuller, 2012). These responses contribute to the ambiguity of the experience because they do not conform with how people *expect* victims to behave. Consistently, people are less inclined to believe victims who do not show the “appropriate” levels of emotional distress following an assault (Schuller et al., 2010). Disambiguating sexual encounters is further complicated by the presence of alcohol and drugs (Abbey, 2002; Untied et al., 2012), as intoxication and memory loss leaves people unclear and uncertain as to whether they have been a willing and consenting participant (Peterson & Muehlenhard, 2004). Disagreements over the requirement of affirmative consent (Humphrey, 2016; Marciniak, 2015), whether intoxication and memory loss equate criminal culpability (Davis & Loftus, 2004), and the importance of who was intoxicated during the act (Maurer, 2016; Wild et al., 1998) pose clear hurdles to overcome when acknowledging sexual assault. The complexity and ambiguity of the social interactions that encompass many sexual assaults and experiences can therefore present additional barriers to people acknowledging that they have been victims of assault. Thus, psychological interventions that make it possible to evaluate interactions more objectively should help people navigate ambiguous sexual encounters.

## Opening the Psychological Toolkit: Perspective Taking & Self-Distancing

Even the act of simply questioning whether you were a victim of sexual violence can be a threatening and disempowering experience. Add to that the fear of reprisals for coming forward, and the barriers to acknowledging a sexual assault quickly escalate. Consequently, people might choose to err on the side of perceived caution—deciding that an experience did not meet the criteria of sexual assault. However, while denial can be useful for avoiding stress and anxiety in the short-term, in the long-run it is an ineffective coping strategy (Suls & Fletcher, 1985). Thus, people need tools that will help them circumvent the stress and anxiety associated with acknowledging that they have been victimized, so that they can label ambiguous encounters more concretely and seek help as appropriate.

One psychological tool that might help in these encounters is taking a self-distanced perspective. Perspective-taking refers to the act of imagining how another person does or would feel in a situation (Kross & Ayduk, 2017), and can result from a manipulated mindset, or capture a trait-level tendency to take a distanced approach to self-reflection (Ayduk & Kross, 2010). People can be encouraged to self-distance and step out of their own mind by imagining they are a neutral third party observing the events or by imagining that the event has happened to someone else entirely (Ayduk & Kross, 2010; Batson et al., 1997). Self-distancing, whether spontaneous or induced, has been linked with positive outcomes following negative interpersonal interactions (Kross & Ayduk, 2017). For example, people who spontaneously imagine conflicts from a third-person perspective report feeling less angry with their conflict partner (Ayduk & Kross, 2010). Similar effects have been shown when people are asked to imagine how they would react to hypothetical experiences or scenarios that *others* have experienced (Coke et al., 1978). People are also better at making rational, wise decisions when they consider them from a distance or imagine them from another person’s perspective because they are less guided by their feelings (Kross & Grossmann, 2012). When it comes to acknowledging sexual assault following ambiguous encounters, perspective-taking might be an effective way of minimizing the negative feelings associated with being a victim—which can lead people to be defensive and use denial—and instead focus on other features of the interaction in order to reach a decision (e.g., state of mind, alcohol use). Thus, imagining the encounters from a friend’s or other distanced perspective might help them label the experience as inappropriate and open them up to the idea of seeking out resources designed to support victims of sexual assault.

## Current Research

The current research aimed to develop a better understanding of the factors contributing to unacknowledged sexual assaults by examining the contextual and psychological factors people use to determine when a sexual assault has or has not occurred following more ambiguous social encounters. To our knowledge, all of the prior research on this topic has either relied on retrospective accounts made by people who acknowledge they have been a victim of assault, or asked people to make determinations based on descriptions of a scenario where someone leaves the sexual encounter claiming to be assaulted or in visible distress. However, the majority of interpersonal encounters are somewhat more ambiguous, especially in sexual contexts where people rely heavily on nonverbal communication. This ambiguity is especially likely when drugs and/or alcohol have been used prior to or during sex, and people might have difficulty fully recollecting the full series of events from the night before. Furthermore, being the victim of a sexual assault can be a negative, threatening, and disempowering experience. People might therefore be more defensive about acknowledging that they themselves have been a victim of assault in ambiguous sexual encounters, rather than if someone else described the same event.

We therefore hypothesized that when people distanced themselves from a sexual experience by taking another person’s perspective, they would evaluate the sexual experience more negatively (i.e., *less* consensual, *more* inappropriate) than when they did not distance from the experience (Hypothesis 1, Studies 1a/1b, 2, and 3). We also tested the extent to which contextual information surrounding the experience (i.e., distress; alcohol consumption/intoxication) helped or hindered people’s ability to determine whether an assault had occurred, and whether this amplified or undermined the effect of perspective taking. We hypothesized that the effect of perspective taking would be particularly beneficial following an ambiguous encounter compared to when clear distress was expressed (Hypothesis 2, Study 2). Furthermore, we hypothesized that alcohol consumption would influence perceptions of the interactions, such that people would evaluate the experience more negatively when only the victim (vs. victim and perpetrator) showed signs of intoxication (e.g., memory loss) and that the ambiguity surrounding alcohol intoxication would interact with perspective taking (Hypotheses 3 and 4, Studies 1a and 1b). We tested these hypotheses in a series of four experimental studies that manipulated perspective taking, and the context under which the events unfolded. Preregistration for each study, study materials, and anonymized data are available on the project’s OSF website (https://osf.io/5bw9h/). Questions containing potentially identifiable responses (e.g., open-ended comments) have been removed to protect participant anonymity. The online supplemental materials also include the instructions, scenarios and writing prompts used in all four studies.

## Studies 1a and 1b

Study 1a examined how both contextual cues (e.g., alcohol consumption; memory of events) and psychological distance (self vs. friend perspective taking) influence how women label ambiguous sexual encounters. Study 1b was a direct replication and extension of Study 1a, with additional questions examining anticipated recovery times and interest in sexual assault resources.

### Methods

#### Participants.

Participants all our studies were recruited using Prolific Academic.^1^1.Prolific Academic was used as the recruitment platform for all four studies. Participants were restricted from taking part in more than one study. Participants in all four studies received the same payment (£1.67 per survey, which is equivalent with £5.00 GBP/hour). Prolific is an online recruitment platform based in the UK, which allows researchers to post links to their studies and pay the participants directly via the platform without needing to exchange personalized or individuated information. Prolific requires researchers pay participants a minimum rate of £5.00GBP/hour. Participants in Studies 1a and 1b therefore received £1.67 for their participation in a 20- minute study. Finally, participants recruited for one study were restricted from taking part in any of the subsequent studies.

Study 1a included 357 British women between the ages of 18 and 24 (*M*_age_ = 21.07, *SD* = 2.00), who identified as heterosexual (77.31%) or bisexual.^2^2.We restricted participation to heterosexual and bisexual women because the potential perpetrator in the vignettes was always a man, and in an interaction, which begins consensually (i.e., in a plausible relationship initiation context). Although sexual assault by women against either women or men is a real possibility, narratives around men as perpetrators are more common. Thus, for the purpose of the current research we relied on these existing sexual assault narratives. Future research should, however, examine the rationalizations lesbian and bisexual women make when the perpetrator is a woman. The majority of the participants identified as white (81.23%), Asian (8.96%), or with mixed or multiple ethnic backgrounds (6.44%). The majority of participants reported that they were current romantically involved (6.44% dating casually; 47.90% dating exclusively; 9.24% engaged or married) and the rest were single (36.41%). Finally, nearly all of our participants reported that they typically practice monogamy (91.60%), although a small percentage said they engage in consensual nonmonogamy/polyamory or another type of relationship style (8.40%).

Study 1b included 423 British women between the ages of 18 and 24 (*M*_age_ = 21.14, *SD* = 2.03), who identified as heterosexual (82.27%) or bisexual. The majority of participants identified as white (90.31%, 3.55% Asian, 2.36% black, 3.07% mixed or multiple ethnic backgrounds). In this sample, 30.50% of participants in the study reported being single/romantically unattached and 57.92% were in an exclusive, committed dating relationship, while the rest were casually dating (6.38%) or engaged or married (5.20%). Finally, the majority of participants reported that they typically practice monogamy (91.49%), although there were still a small number who indicated that engaged in consensual nonmonogamy/polyamory or another type of relationship style (8.51%).

#### Materials and Procedures.

In Study 1a, participants were instructed to imagine four sexual encounters. Half the participants were instructed to imagine they had experienced the encounters and were describing them to a close friend (own-perspective condition), and half of the participants were instructed to imagine a close friend was describing the encounters to them (other-perspective condition). Within-subjects, each scenario included a contextual cue regarding the actors’ intoxication during the sexual encounter (two-levels; both man and woman intoxicated vs. only the woman intoxicated) and how much the woman could recall following the encounter (two-levels; no memory vs. full memory). Participants were then asked to indicate how they perceived the man’s and woman’s interest and sexual enjoyment during the encounter, whether the man had behaved coercively or inappropriately, and how positively (7-items; 1 = not at all, 7 = extremely) and negatively (7-items; 1 = not at all, 7 = extremely) the woman in the scenario would have felt. Participants were also asked to decide whether consent had been obtained (1 = yes, 0 = no), and whether the encounter described a rape (1 = yes, 0 = no). Following the vignettes, participants completed a single-item measure of spontaneous self-distancing (1 = predominantly experienced through own eyes, 7 = predominantly experienced as an observer; Ayduk & Kross, 2010), which was included in the analyses to account for trait-level individual differences in the tendency to spontaneously engage in distanced perspective taking.

Study 1b followed the same procedures of Study 1a, with the addition of asking participants how long it would take them/their friend to recover from the experience described in the scenario (1 = will recover very quickly, 7 = will take a long time to recover), and whether they would be interested in/recommend information about available sexual assault resources following the experience (1 = not at all, 7 = extremely). Furthermore, the two questions assessing whether consent had been obtained in the scenario and whether it described a rape where changed from a binary “yes/no” outcome to a continuous “1 = definitely did not; 7 = definitely did”. This change was made following Study 1a in order to try and increase the potential for variability in how people make these decisions. See the online supplemental materials for the full descriptions of the measures.

### Results and Discussion

#### Perspective Taking.

First, we tested whether perspective taking (–1 = imagine friend, 1 = imagine self) influenced perceptions of the sexual encounters collapsing across all 4 scenarios and controlling for individual differences in spontaneous self-distancing (Table 1). In both studies, when women were told to imagine the scenarios from their own perspective, they were more likely to say that the man was less interested in having sex (Study 1a: *b* = –.13, *t*(354) = –2.40, *p* = .02, *η*^2^_partial_ = .01; Study 1b: *b* = –.12, *t*(420) = –2.47, *p* = .01, *η*^2^_partial_ = .01), his behavior was less inappropriate (Study 1a: *b* = –.14, *t*(354) = –2.37, *p* = .02, *η*^2^_partial_ = .01; Study 1b: *b* = .002, *t*(420) = –.03, *p* = .98, *η*^2^_partial_ < .001), and less coercive (Study 1a: *b* = .13, *t*(354) = –1.90, *p* = .047, *η*^2^_partial_ = .01; Study 1b: *b* = –.06, *t*(420) = –1.04, *p* = .30,, *η*^2^_partial_ = .003), than women who imagined the scenarios from a friend’s perspective. Women who imagined themselves in the scenarios also reported that they would have experienced more negative affect (Study 1a: *b* = .14, *t*(354) = 2.34, *p* = .02, *η*^2^_partial_ = .02; Study 1b: *b* = .10, *t*(420) = 1.95, *p* = .052, *η*^2^_partial_ = .02), and less positive affect, *b* = .24, *t*(354) = –4.94, *p* < .001, *η*^2^_partial_ = .07; Study 1b: *b* = –.23, *t*(420) = –5.27, *p* < .001, *η*^2^_partial_ = .08), in the encounters than women who imagined a friend. This suggests that women were likely not trivializing or discounting the impact of the scenarios when they imagined themselves, but instead might be defensively reacting against the anticipated negative emotions these ambiguous sexual situations might elicit. However, contrary to our expectations, there were no differences across perspective taking condition predicting whether consent had been given (Study 1a: *b* = –.01, *t*(354) = –.65, *p* = .51, *η*^2^_partial_ = .001; Study 1b: *b* = .001, *t*(420) = .02, *p* = .99, *η*^2^_partial_ < .001), or whether the scenario described a rape (Study 1a: *b* = .01, *t*(354) = .40, *p* = .69, *η*^2^_partial_ = .001; Study 1b: *b* = –.03, *t*(420) = –.41, *p* = .68, *η*^2^_partial_ < .001). This suggests that there is potentially a disconnect between labeling sexual encounters as coercive or inappropriate and labeling them as nonconsensual. We return to these issues in the general discussion.

**Table 1.** Model Coefficients for Effects of Perspective Taking Condition for Study 1a and 1b

	Study 1a				Study 1b			
	Main Effect: Perspective Condition		Covariate: Spontaneous Self-distancing		Main Effect Perspective Condition		Covariate: Spontaneous Self-distancing	
	*b*	*t*	*b*	*t*	*b*	*t*	*b*	*t*
Woman wanted sex	–.06	–1.18	–.04	–1.59	–.07	–1.34	–.03	–1.11
Man wanted sex	–.13	–2.40*	–.06	–2.39*	–.12	–2.47*	–.01	–.56
Sex mutually satisfying	–.03	–.5	–.02	–.66	–.01	–.24	.02	.81
Man inappropriate	–.14	–2.37*	–.02	–.63	–.002	–.03	–.005	–.18
Man coercive	–.13	–1.99*	–.04	–1.38	–.06	–1.04	.004	.13
Negative affect	.14	2.34*	.01	.26	.1	1.95†	–.04	–1.64
Positive affect	–.24	–4.94***	–.01	–.51	–.24	–5.27***	.04	1.85†
Recovery					.08	1.27	–.01	–.45
Resources					–.34	–5.18***	–.04	–1.43
Consent given	–.01	–.65	–.005	–.72	.001	.02	.01	.55
Was rape	.01	.4	–.004	–.57	–.03	–.41	.001	.3

*Note.* †*p* < .10. *p < .05. **p <. 01. ***p < .001.

Finally, in Study 1b, perspective taking did not predict differences in the amount of time people thought would be needed for recovery, *b* = .08, *t*(420) = 1.22, *p* = .21, *η*^2^_partial_ = .01. However, it did predict interest in (imagine-self condition), or willingness to recommend (imagine-friend condition), sexual assault resources, *b* = –.34, *t*(420) = –5.18, *p* < .001, *η*^2^_partial_ = .06. Relative to women who imagined the scenarios happening to a friend, women who imagined themselves in the scenarios, said they would be less interested in sexual support resources, despite more negative feelings towards the encounters.

#### Contextual Cues.

Next, we used repeated measures ANOVAs to test the within-subject’s effects of contextual cues and whether these cues interacted with perspective taking. Perspective taking condition did not interact with scenario to influence how women evaluated the hypothetical ambiguous sexual encounters. However, different combinations of intoxication and memory loss significantly contributed to how women evaluated the scenarios across all measures (*ps* < .001) with the exception in Study 1a of whether or not the man wanted to have sex, *F*(1,357) = .68, *p* = .43, *η*^2^_partial_ = .002 (Tables 2 and S1 & S2). Planned pairwise comparisons between scenarios demonstrated that women had more positive perceptions of the events when both the man and the woman were described as being intoxicated, compared to when only the woman was described as being intoxicated, and when the woman had full memory of night’s events instead of no recollection (see Figures S1 and S2 in Online Supplemental Material). Additionally, scenarios in which only the woman was described as intoxicated and could not remember the encounter were evaluated the most negatively across participants. This suggests that while taking a different perspective might be useful to help compensate for expected negative feelings following a sexual encounter, women might also use contextual cues to help determine the severity of the situation when dealing with otherwise ambiguous information about a social interaction. Furthermore, in Study 1b scenario and perspective-taking condition interacted to predict whether people would recommend or be interested in looking into sexual assault resources following the encounter, *F*(1, 422) = 13.17, *p* < .001, *η*^2^_partial_ = .03. In every scenario, except when the both people were intoxicated and had full memory (*M*_self_ = 1.68 (*SD* = 1.12), *M*_friend_ = 1.89 (*SD* = 1.17); *t*(427) = –1.84, *p* = .07, 95% CI[–.42, .01]), women were more likely to say they would recommend looking into resources when they imagined a friend in the scenario than themselves (*ts* > –4.41, *ps* < .001).

Findings from Study 1a and 1b suggest that people use both psychological (i.e., perspectives) and contextual (i.e., alcohol; memory) information separately to make determinations about ambiguous sexual experiences. When women were presented with four scenarios describing an ambiguous sexual encounter, they were more likely to say that the man’s behaviors had been inappropriate or coercive when they imagined their friend in the situation than themselves. However, they also anticipated feeling worse following the sexual experiences than women who imagined a friend. Furthermore, Study 1b also showed that despite anticipating more negative emotions following the experiences, women were less interested in sexual assault resources when imagining the scenarios had happened to themselves compared to a friend. These findings are consistent with women experiencing negative consequences associated with unacknowledged sexual assaults, while still labeling the encounters as consensual or not inappropriate. Furthermore, the findings from these studies suggest that contextual cues, such as who was intoxicated and how much could be remembered, might be heavily relied on to help determine whether something inappropriate had occurred, rather than how women feel about the encounter.

**Table 2.** Mean Ratings across Scenarios in Studies 1a and 1b

	Scenario 1		Scenario 2		Scenario 3		Scenario 4		*F*	
	Both Drunk, Full Memory		Both Drunk, No Memory		Woman Drunk, Full Memory		Woman Drunk, No Memory			
	*M1a*(*SD*)	*M1b*(*SD*)	*M1a*(*SD*)	*M1b*(*SD*)	*M1a*(*SD*)	*M1b*(*SD*)	*M1a*(*SD*)	*M1b*(*SD*)	Study 1a	Study 1b
Woman wanted sex	5.48(.07)^b,c,d^	5.54(.06)^b,c,d^	2.9(.08)1^a,c,d^	2.78(.07)^a,c^	4.56(.08)^a,b,d^	4.65(.08)^a,b,d^	2.64(.07)^a,b,c^	2.69(.07)^a,c^	592.91***	655.28***
Man wanted sex	5.57(.07)^b^	5.64(.06)^b^	4.94(.08)^a,c,d^	4.93(.07)^a,c,d^	5.51(.07)^b^	5.57(.06)^b^	5.46(.07)^b^	5.59(.07)^b^	.68	4.20*
Sex mutually satisfying	5.62(.07)^b,c,d^	5.58(.06)^b,c,d^	2.99(.09)^a,c,d^	2.95(.07)^a,c,d^	3.92(.08)^a,b,d^	3.91(.08)^a,b,d^	2.41(.07)^a,b,c^	2.36(.06)^a,b,c^	939.79***	974.21***
Man inappropriate	2.51(.08)^b,c,d^	2.57(.07)^b,c,d^	4.39(.09)^a,d^	4.62(.08)^a,d^	4.49(.10)^a,d^	4.47(.09)^a,d^	5.41(.08)^a,b,c^	5.58(.07)^a,b,c^	678.83***	852.76***
Man coercive	2.38(.08)^b,c,d^	2.39(.06)^b,c,d^	4.05(.09)^a,d^	4.18(.08)^a,d^	4.14(.10)^a,d^	4.14(.09)^a,d^	4.96(.09)^a,b,c^	4.94(.08)^a,b,c^	552.28***	606.14***
Negative affect	2.60(.07)^b,c,d^	2.59(.06)^b,c,d^	4.18(.08)^a,c,d^	4.42(.07)^a,c,d^	3.96(.07)^a,b,d^	4.17(.07)^a,b,d^	4.83(.07)^a,b,c^	4.84(.07)^a,b,c^	649.04***	720.41***
Positive affect	3.93(.07)^b,c,d^	3.84(.07)^b,c,d^	2.41(.06)^a,c,d^	2.23(.05)^a,c,d^	2.80(.06)^a,b,d^	2.62(.06)^a,b,d^	2.08(.05)^a,b,c^	2.02(.05)^a,b,c^	621.38***	611.27***
Recovery		2.82(.08)^b,c,d^		4.39(.08)^a,c,d^		4.02(.08)^a,b,c^		5.11(.08)^a,b,c^		594.02***
Resources		1.78(.06)^b,c,d^		3.17(.08)^a,c,d^		2.76(.08)^a,b,d^		3.70(.09)^a,b,c^		478.05***
Consent given	.80(.02)^b,c,d^	5.13(.08)^b,c,d^	.24(.02)^a,c,d^	2.50(.07)^a,c,d^	.58(.03)^a,b,d^	3.88(.09)^a,b,d^	.07(.01)^a,b,c^	1.88(.06)^a,b,c^	709.63***	1018.92***
Was rape	.05(.01)^b,c,d^	2.03(.06)^b,c,d^	.46(.03)^a,c,d^	4.24(.09)^a,c,d^	.31(.02)^a,b,d^	3.52(.09)^a,b,d^	.77(.02)^a,b,c^	5.16(.08)^a,b,c^	711.98***	1047.77***

*Note:* ^†^*p* < .10. *p < .05, **p < .01, *** *p* < .001. Significant pairwise comparisons (*p* < .05) are denoted as follows:

^a^differs from Scenario 1, ^b^differs from Scenario 2, ^c^differs from Scenario 3, and ^d^differs from Scenario 4.

## Study 2

The aim of Study 2 was to extend the findings from Studies 1a and 1b by examining the extent to which people rely on clear distress signals when acknowledging sexual assault, and whether this varies as a function of perspective taking.

### Methods

#### Participants.

Four hundred and fifty-one British women between the ages of 18 and 24 (*M*_age_ = 21.00, *SD* = 1.97) were recruited via Prolific to take part in a 20-minute survey in exchange for £1.67. The sample was similar demographically to Studies 1a and 1b: The majority (82.93%) of the sample identified as white (5.54% Asian, 6.87% mixed or multiple ethnic backgrounds, 2.22% black, 2.43% middle eastern/latinx/other ethnicity not listed), heterosexual (79.60%), in a relationship (54.99% exclusively dating, 5.32% casually dating, 6.43% engaged/married, 33.26% single or separated), and monogamous (90.91%).

#### Materials and Procedures.

Participants were presented with a single scenario originally from the previous studies and asked to evaluate the scenario with the same questions from Studies 1a and 1b. However, in addition to randomly assigning participants to the self-perspective or friend-perspective conditions, participants were also randomly presented with evidence of distress or ambiguity. In the ambiguity condition, participants were presented with the scenario as presented in Studies 1a/1b. In the distress condition, a final sentence was added to the scenario description to establish the woman had concerns about the experience (i.e., “I feel sick to my stomach thinking about it.”).

### Results and Discussion

We used linear regressions to predict the same evaluations from Studies 1a/1b from the (a) main effects of perspective-taking condition (–1 = friend, 1 = self), and distress condition (–1 = ambiguous, 1 = distressed), and (b) their two-way interaction, controlling for spontaneous self-distancing (centered) (Table 3).

**Table 3.** Model Coefficients for Study 2

	Woman Wanted Sex		Man Wanted Sex		Sex Mutually Satisfying		Man Inappropriate		Man Coercive		Negative Affect		Positive Affect		Recovery		Resources		Consent Given		Was Rape	
Predictors	*b*	*t*	*b*	*t*	*b*	*t*	*b*	*t*	*b*	*t*	*b*	*t*	*b*	*t*	*b*	*t*	*b*	*t*	*b*	*t*	*b*	*t*
Perspective taking condition	.06	.35	–.11	–.57	.10	.49	–.16	–.68	–.13	–.58	.62	3.68***	–.45	–3.10**	.66	3.16**	–.41	–2.10*	.10	.50	–.05	–.24
Distress condition	–.48	–2.90**	–.32	–1.64	–.70	–3.53***	–.08	–.35	–.09	–.40	1.18	7.12***	–.65	–4.59***	.60	2.91**	.85	4.41***	–.47	–2.38*	.12	.55
Perspective × distress	.02	.09	.37	1.33	–.15	–.53	.39	1.21	.30	.94	–.67	–2.83**	.23	1.13	–.22	–.75	–.69	–2.52*	–.19	–.68	.28	.91
Spontaneous self-distancing	.01	.29	–.01	–.37	–.01	–.31	.07	1.62	.06	1.62	–.01	–.44	.01	.36	.05	1.50	.03	.98	–.002	–.07	.01	.13

*Note:* †*p* < .10. *p < .05, **p < .01, ***p < .001.

**Main Effect of Perspective Taking**. Significant main effects of perspective-taking condition emerged predicting greater negative affect, *b* = .62, *t*(446) = 3.68, *p* < .001, *η*^2^_partial_ =.01, lower positive affect, *b* = –.45, *t*(446) = –3.10, *p* = .002, *η*^2^_partial_ =.02, faster anticipated recovery, *b* = .66, *t*(446) = 3.16, *p* = .002, *η*^2^_partial_= .03, and lower interest in resources, *b* = –.41, *t*(446) = –2.10, *p* = .04, *η*^2^_partial_ =.07, when women imagined themselves compared to a friend.

**Main Effect of Clear Distress.** Significant main effects of distress emerged predicting lower interest in having sex for the woman, *b* = –.48, *t*(446) = –.2.90, *p* = .004, *η*^2^_partial_= .03, lower mutual sexual satisfaction, *b* = –.70, *t*(446) = –3.53, *p* = .0005, *η*^2^_partial_ = .06, slower anticipated recovery, *b* = .60, *t*(446) = 2.91, *p* = .004, *η*^2^_partial_ = .03, greater interest in resources, *b* = .85, *t*(446) = 4.41, *p* < .001, *η*^2^_partial_ = .03, greater negative affect, *b* = 1.18, *t*(446) = 7.12, *p* < .001, *η*^2^_partial_ = .10, and lower positive affect, *b* = –.65, *t*(446) = –4.59, *p* < .001, *η*^2^_partial_ = .06. Interestingly, although no differences emerged across evaluations of inappropriateness, coerciveness, or whether the scenario qualified as a rape (*ps* > .55), people in the distressed compared to ambiguous condition believed it was less likely that the woman had given consent in the scenario, *b* = –.47, *t*(446) = –2.38, *p* = .02, *η*^2^_partial_ = .04.

**Two-way Interaction.** The two-way perspective-taking by distress condition interaction was only significant for negative mood and interest in resources (Figure S3). We decomposed the interaction by looking at the simple main effect of perspective-taking condition for people in the distressed and ambiguous conditions. When distress was expressed, there was no significant main effect of perspective-taking predicting anticipated negative affect in the scenario, *b* = –.04, *t*(446) = –.48, *p* = .80, *η*^2^_partial_ = .01. However, when the scenario was ambiguous, women anticipated feeling more negatively following the scenario when they imagined themselves compared to a friend, *b* = .62, *t*(446) = 3.68, *p* < .001, *η*^2^_partial_ = .01.

When it came to interest in sexual assault resources, the simple effect of target was significant for both women in the distressed, *b* = –1.10, *t*(446) = –5.70, *p* < .001, *η*^2^_partial_ = .07 and ambiguous conditions, *b* = –.41, *t*(446) = –2.10, *p* = .04, *η*^2^_partial_ = .07, although the difference was somewhat attenuated for ambiguous sexual encounters. This suggests that women were actually somewhat more interested in resources following an ambiguous sexual encounter, perhaps tapping into a desire for more information when people are trying to make sense of their experiences. However, women in general were still significantly less likely show an interest in these types of resources for themselves compared to a friend.

Consistent with the findings from Studies 1a/1b, women were more likely to discount the negativity of an ambiguous sexual encounter and were more reticent to seek support resources when they imagined themselves than their friends, even though they believed it would take themselves longer to recover and they would feel more negatively about the experience afterwards. Despite evidence from Studies 1a/1b that women anticipate strong negative emotions following ambiguous sexual encounters, explicit evidence of distress made women evaluate the sexual scenario more negatively as one might expect. However, even with this explicit assertion of concern about the encounter, and greater endorsement that these encounters were less wanted, less enjoyable and less consensual for the woman, people still failed to acknowledge them as a sexual assault. We return to this point in the general discussion.

## Study 3

Finally, the aim of Study 3 was to extend the findings from Studies 1a/1b and 2 from hypothetical scenarios and apply them to women’s real-life experiences. Thus, Study 3 tested whether instructing women to describe *actual* sexual encounters from a first versus third person perspective would influence their perceptions of the encounters and willingness to engage with sexual assault resources. Women were specifically asked to write about their worst sexual experience. There were several motivations behind asking women to describe their “worst” sexual experience rather than explicitly requesting they describe a sexual assault. First, the results from Studies 1a, 1b, and 2 suggested that defining a sexual encounter as an assault is challenging from multiple perspectives – a lack of consent or interest in the encounter were not necessarily enough for women to label encounter as an assault. Furthermore, the research on unacknowledged sexual assault suggests that many women can describe sexual experiences that meet the hallmarks of an assault but would not label them as such. Thus, in order to get as much variability in experience as possible, we intentionally left the sexual encounter open to interpretation on the part of the participant. This meant that participants described experiences that ranged from a dissatisfying, yet fully consensual, sexual encounter, through to acknowledged sexual assaults. Furthermore, in order to avoid priming participants with expectations of what does/does not qualify as a bad or nonconsensual sexual experience, participants were not asked to reflect on the ambiguity of the encounter, the clarity of their memory for the details of the encounter, or whether anyone involved was intoxicated. This made Study 3 different from the previous studies in two important ways: (1) participants were reflecting on real experiences, and (2) we were not able to speak to the added importance of contextual cues above and beyond self-distancing. Nonetheless, this study provides an important test of the role of self-distancing in the acknowledgment of sexual assault.

### Methods

**Participants.** Three hundred and seventy-eight British women between the ages of 18 and 24 (*M*_age_ = 21.08, *SD* = 1.98), were recruited via the Prolific platform to take part in a 20-minute survey in exchange for £1.67. Again, demographics were consistent with the other studies: the majority of participants were white (88.10%, 5.03% Asian, 5.03% mixed/multiple ethnic backgrounds, 2.12% black/latinx/other ethnicity not listed), heterosexual (73.02%), in a relationship (60.85% exclusively dating, 7.94% casually dating, 6.35% engaged/married, 25.13% single or separated), and monogamous (93.12%).

**Materials and procedures.** Participants were asked to remember their worst sexual experience and write about it either from a first-person perspective or third-person perspective (Kross et al., 2014). Participants were then asked to evaluate the sexual encounter with the same measures as in Study 2.

## Results and Discussion

Linear regressions were used to predict the same scenario outcomes from the previous studies from the main effect of perspective-taking condition (–1 = friend, 1 = self) and the centered effect of spontaneous self-distancing. Contrary to the hypotheses and findings from the previous studies, perspective-taking condition was not significantly associated with any of the outcomes measured when participants were asked to reflect on their own actual sexual experiences instead of a hypothetical one (*ps* > .33). However, a significant main effect of individual differences in spontaneous self-distancing was significant across outcome measures (Table 4). Furthermore, unlike in Studies 1a/1b–2, the perspective taking condition was not significantly associated with spontaneous self-distancing, *b* = –.27, *t*(377) = –1.33, *p* = .19, η^2^_partial_ = .005, suggesting that while the writing prompt did not help women view their previous sexual encounters from a distanced perspective, women varied in their individual tendency to recollect their personally experienced event from a distanced perspective. Thus, we explored how spontaneous self-distancing influenced evaluations of self-ascribed worst sexual experiences.

**Table 4.** Model Coefficients for Study 3

	Perspective Condition		Spontaneous Self-distancing	
	*b*	*t*	*b*	*t*
Woman wanted sex	.17	.86	–.15	–3.06**
Man wanted sex	–.16	–.98	–.11	–2.70**
Sex mutually satisfying	.12	.77	–.09	–2.39*
Man inappropriate	–.12	–.54	.17	2.89**
Man coercive	.03	.13	.17	3.19**
Negative affect	.09	.62	.07	1.91†
Positive affect	.10	.84	–.07	–2.12*
Recovery	.01	.05	.17	3.38***
Resources	.06	.46	.10	2.98**
Consent given	.04	.22	–.19	–3.82***
Was rape	–.02	–.11	.18	4.02***

*Note*: †*p* < .1, *p < .05, **p < .01, ***p < .001.

When women reported that they spontaneously recalled their worst sexual experience from the eyes of an observer, they were more likely to report that both the man, *b* = –.11, *t*(376) = –2.70, *p* = .01, η^2^_partial_ = .02, and the woman, *b* = –.15, *t*(376) = –3.06, *p* = .002, η^2^_partial_ = .02, were less interested in having sex, the sex was less mutually satisfying, *b* = –.09, *t*(376) = –2.39, *p* = .02, η^2^_partial_ = .02, that the man had behaved inappropriately, *b* = .17, *t*(376) = 2.98, *p* = .003, η^2^_partial_ = .02, and coercively, *b* = .17, *t*(376) = 3.19, *p* = .002, η^2^_partial_ = .03, that they were less likely to have given consent, *b* = –.19, *t*(376) = –3.82, *p* < .001, η^2^_partial_ = .04, that the experience could be defined as a sexual assault, *b* = .18, *t*(376) = 4.02, *p* < .003, η^2^_partial_ = .04, and that they expected a longer recovery period, *b* = .17, *t*(376) = 3.38, *p* < .001, η^2^_partial_ = .03, compared to when they imagined it from their own perspective. Likewise, women who recalled the experience as a distanced observer were more interested in learning more about sexual assault resources than those who recalled them from their own perspective, *b* = .10, *t*(376) = 2.98, *p* = .003, η^2^_partial_ = .02.

Notably, the effects of spontaneous self-distancing on recalled affect during the experience did not conceptually replicate the effects of affect from the previous studies. Instead, greater spontaneous self-distancing was associated with greater, albeit not significantly, negative affect during the encounter, *b* = .07, *t*(376) = 1.91, *p* = .06, η^2^_partial_ = .01, and significantly less positive affect, *b* = –.07, *t*(376) = –2.12, *p* = .03, η^2^_partial_ = .01, compared to those recalling from their own perspective. However, this difference might also reflect differences in anticipating how one *might* feel in an experience compared to how one *actually* felt, or because participants were asked to recall their *worst* sexual encounter as opposed to an ambiguous one.

Crucially, spontaneous self-distancing was associated with whether people felt consent had been obtained during the sexual experience, *b* = –.19, *t*(376) = –3.82, *p* < .001, η^2^_partial_ = .04, and whether the experience could be labeled a sexual assault, *b* = .18, *t*(376) = 4.02, *p* < .001, η^2^_partial_ = .04. Thus, consistent with the previous studies, women who had the tendency to recall their worst sexual experience from the eyes of an observer were more likely to say consent had not been given and that the experience could have been considered a sexual assault.

Perspective taking manipulations might not help women retrospectively acknowledge or accept inappropriate sexual experiences and seek help. However, the extent to which women spontaneously engaged in self-distancing—the trait-level ability to automatically view experiences from a distanced perspective and engage in adaptive self-reflection (Ayduk & Kross, 2010)—seemed to help women label sexual experiences as more inappropriate, coercive, and nonconsensual, and needing more time to recover from. This difference of perspective also helped women feel more open to seeking resources aimed at supporting sexual assault victims.

## General Discussion

The goal of this research was to better understand the social and psychological processes that contribute to acknowledging sexual assault and whether the psychological process of self-distancing could be leveraged into helping women seek out resources associated with supporting victims of sexual assault. Findings from the studies suggest that taking another person’s perspective can help women acknowledge that a sexual experience has been inappropriate or coercive, and that it might take longer to recover from the experience. Furthermore, taking a distanced perspective was associated with a greater willingness to seek out resources associated with sexual assault and support. However, these are effects were strongest when the person spontaneously engaged in self-distancing rather than when perspective-taking was manipulated, at least when comparing hypothetical experiences against actual past experiences. This research also suggests that women rely on contextual information when determining whether a sexual experience was inappropriate, including contextual information such as alcohol use and intoxication, as well as emotional information such as distress.

The findings from this study are novel in that they asked the participants to make attributions and acknowledge the potential for sexual assault in the description of relatively ambiguous sexual encounters, rather than reflecting on experiences describing violent attacks, the use of coercion, or feelings of guilt or regret, as has commonly been the case (Deming et al., 2013). Although understanding how people come to terms with experiences they acknowledge as sexually violent is vitally important, the current study reflects the more nuanced and complex ways in which people tend to experience sexual encounters. Because people often rely heavily on nonverbal communication to signal the presence and absence of consent, and because many people engage in sex under the influence of drugs and alcohol, people do not always know how to make sense of their sexual experiences (Muehlenhard et al., 2016). Our research suggests that while people will use contextual cues to help fill in the gaps, they are also more likely to believe that a sexual encounter was untoward when they imagine it happening to another person. Furthermore, we found that women who spontaneously think about their own, *actual* sexual experiences from a distanced perspective were more open to acknowledging that the experience was nonconsensual, that it was likely a sexual assault, and were also more open to learning about sexual assault resources. Future research should examine whether people can be trained to use spontaneous self-distancing strategies so that it becomes something that women engage in automatically when they reflect on their sexual histories. This might make it possible for more women to acknowledge their experiences as instances of sexual violence and to consequently seek out important health and social support resources.

Finally, the findings from this research highlight the importance of understanding the factors that lead people to acknowledge and label a sexual experience as an assault. Consistent with research on unacknowledged sexual assault (Littleton & Axsom, 2003; Littleton et al., 2007), we found that women in our studies were very reluctant to say an assault had occurred, even when they believed the encounters were inappropriate, coercive, and even nonconsensual. This suggests a continued disconnect between lay understandings or interpretations of sexual assault and legal definitions (Haugen et al., 2018; Pugh & Becker, 2018). The silver lining to these findings is that despite this reluctance to label an experience as an assault, people were motivated to learn more about sexual assault resources when the situations were otherwise rated more negatively. These resources might therefore offer a gateway through which to learn more about nonconsensual sexual experiences. Overall, the findings from this project point to a clear need for continued research on definitions of consent and sexual assault so that better interventions can be developed to help people navigate sexual encounters in a healthy way, and to feel equipped to label their experiences in a way that will empower them to seek help and support in the future.

## Limitations and Future Directions

One notable set of finding from Studies 1a and 1b is that alcohol use and memory loss were important cues that something inappropriate had transpired. This was especially the case when only the woman in the scenario was intoxicated. These findings somewhat contradict existing research on victim-blaming, where a victim’s intoxication is used as justification or dismissal of his or her attack (Richardson & Campbell, 1982). These findings suggest that people recognize that alcohol can be used to ply or take advantage of another person, even in an ambiguous context where the possible victim does not show any signs of distress the next day. This study did not test victim blaming or culpability on the part of the ostensible victim. Future research should examine how alcohol, endorsement of inappropriateness or coercion, and victim-blaming interact across different sexual experiences. For example, it is possible that people acknowledge that extreme intoxication limits a woman’s ability to consent and casts the man’s actions in a bad light, but that they also feel that the woman is equally culpable for reaching that state of intoxication volitionally. Our findings also suggest that alcohol intoxication is not viewed as negatively when both sexual partners are intoxicated, suggesting that shared state of mind or equal footing (vs. implied power imbalances) is an important factor people take into consideration when acknowledging sexual assault.

Another limitation of the current studies is that they focus on heterosexual and bisexual women’s experiences with men. This restriction was for the purpose of developing believable sexual encounters for Studies 1–3 that reflect how young adults behave in casual sexual interactions. However, women can also be perpetrators of sexual assault, despite rape myths which suggest otherwise (Chapleau et al., 2008). Thus, future research should replicate and extend the current findings to ensure that they apply equally across sexual orientations and for both male and female perpetrators.

Finally, the current research was restricted to young adults between the ages of 18 and 24. One reason for this restriction was pragmatic, in that we wanted to create hypothetical encounters that reflect typical dating and hook-up norms for participants. However, we also chose to restrict to this age group because they have the highest reported incident rates of sexual assaults (Department of Justice, 2014). However, norms regarding consent, sexual assault and victim blaming have shifted substantially over time, especially in the past five to 10 years with movements such as #MeToo (Forbes, 2018; Time’s Up, 2018). These changes in social awareness and social narratives might mean that women from older generations have been particularly susceptible to the processes that lead to unacknowledged sexual assault. Thus, additional research should examine whether the same contextual and psychological cues predicting acknowledgment of sexual assault emerge across age groups.

## Diversity

Our samples included women living in the United Kingdom. This is unique, as the majority of the research on unacknowledged sexual assault has been completed using college samples in the United States. Although we did not recruit a nationally representative sample, our samples were consistent with the 2011 Census that reports approximately 80% of people in England and Wales identify as white, 7% as Asian, and 3% as Black. However, because the nonwhite samples are relatively small in each study, it is not possible to test for cultural and/or ethnic differences. Our sample also includes people who are single and romantically attached, as well as people who engage in different types of relationship styles (e.g., monogamy, consensual nonmonogamy, polyamory). As noted above, we had pragmatic reasons for restricting participation to women between the ages of 18 and 24, and women who identified as heterosexual or bisexual. However, these restrictions mean that some caution should be used when generalizing our findings across people of different ages.

## Conclusions

Making sense and meaning of experiences can be complicated, and sexual encounters are no exception. Despite clear-cut lay definitions of consent, in practice a lot of people find themselves trying to make sense of unclear or ambiguous sexual encounters that do not fit the traditional narratives surrounding sexual assault. Consequently, when sexual assault occurs, it might be unacknowledged, preventing the victim from accessing the support and resources they need to properly heal. Our findings suggest that in addition to contextual cues (e.g., memories, intoxication), taking a distanced perspective can help women acknowledge inappropriateness in their sexual experiences and a desire to seek help and support.

## Supplemental Material

Supplemental material for this article is available online. Click here for additional data file. Supplemental Material for It Happened to a Friend of Mine: The Influence of Perspective-taking on the Acknowledgment of Sexual Assault Following Ambiguous Sexual Encounters by Veronica M. Lamarche, Laurie James-Hawkins, in Journal of Interpersonal Violence

## Author Biographies

**Veronica Lamarche,** PhD, is a social-personality psychologist and lecturer (assistant professor) in the Department of Psychology at the University of Essex. Her research focuses on the psychological mechanisms associated with balancing risk, vulnerability, trust, and dependence in relationships.

**Laurie James-Hawkins,** PhD, is a lecturer (assistant professor) in the Department of Sociology at the University of Essex. Her research focuses on the intersection of health, gender, and sexuality.
