# Supplementary material for: It Happened to a Friend of Mine: The Influence of Perspective-taking on the Acknowledgment of Sexual Assault Following Ambiguous Sexual Encounters
Source: J Interpers Violence. 2020 Sep 29;37(9-10):NP7343–68. doi: 10.1177/0886260520957678 (PMC9092921; doi:10.1177/0886260520957678)
Supplement: Supplemental material for this article is available online. [file sj-pdf-1-jiv-10.1177_0886260520957678.pdf]

TABLE OF CONTENTS

Supplemental tables & figures .... pg. 2

Scenario instructions & writing prompts .... pg. 6

SUPPLEMENTAL TABLES & FIGURES.

Table S1. Test of Within-Subjects Effects for Study 1a

		Sums of Squares	df	Mean Square	F	$\eta^2_{partial}$
Woman Wanted Sex	Scenario	845.17	1	845.17	592.91***	.62
	Scenario x Target	.52	1	.52	.37	.001
	Error	508.89	357	1.42		
Man Wanted Sex	Scenario	.83	1	.63	.68	.002
	Scenario x Target	.27	1	.27	.20	.66
	Error	474.06	357	1.33		
Sex Mutually Satisfying	Scenario	1353.62	1	1353.62	939.79***	.72
	Scenario x Target	.27	1	.27	.666	.001
	Error	514.20	357	1.44		
Man Inappropriate	Scenario	1393.42	1	1393.42	678.83***	.66
	Scenario x Target	.04	1	.04	.02	.000
	Error	732.81	357	2.05		
Man Coercive	Scenario	1102.64	1	1102.64	552.28***	.61
	Scenario x Target	.07	1	.07	.04	.000
	Error	712.76	357	2.00		
Negative Affect	Scenario	748.46	1	748.46	649.04***	.65
	Scenario x Target	.003	1	.003	.002	.000
	Error	410.53	357	1.15		
Positive Affect	Scenario	476.74	1	476.74	621.38***	.67
	Scenario x Target	1.94	1	1.94	2.52	.01
	Error	273.14	357	.77		
Consent Given	Scenario	60.45	1	60.45	709.63***	.67
	Scenario x Target	.003	1	.003	.04	.000
	Error	30.33	357	.08		
Was Rape	Scenario	70.79	1	70.79	711.98***	.67
	Scenario x Target	.0000003	1	.0000003	.000	.000
	Error	35.40	357	.10		

Note. †$p<0.1$ *$p<.05$ **$p<.01$ ***$p<.001$

Table S2. Test of Within-Subjects Effects for Study 1b

		Sums of Squares	df	Mean Square	F	$\eta^2_{partial}$
Woman Wanted Sex	Scenario	945.78	1	945.78	655.28***	.61
	Scenario x Target	.83	1	.83	.58	.001
	Error	609.09	422	1.44		
Man Wanted Sex	Scenario	5.10	1	5.10	4.20*	.01
	Scenario x Target	.37	1	.37	.30	.001
	Error	512.53	422	1.22		
Sex Mutually Satisfying	Scenario	1600.46	1	1600.46	974.21***	.70
	Scenario x Target	1.38	1	1.38	.84	.002
	Error	693.27	422	1.64		
Man Inappropriate	Scenario	1670.72	1	1670.72	852.76***	.67
	Scenario x Target	1.70	1	1.70	.35	.002
	Error	826.78	422	1.96		
Man Coercive	Scenario	1229.53	1	1229.53	606.14***	.59
	Scenario x Target	6.40	1	6.40	3.16†	.01
	Error	856.02	422	2.03		
Negative Affect	Scenario	897.62	1	897.62	720.41***	.63
	Scenario x Target	.39	1	.39	.31	.001
	Error	524.56	422	1.25		
Positive Affect	Scenario	543.77	1	543.77	611.27***	.59
	Scenario x Target	3.40	1	3.40	3.82†	.01
	Error	374.51	422	.89		
Recovery	Scenario	896.80	1	896.80	594.02***	.58
	Scenario x Target	4.20	1	4.20	2.78†	.01
	Error	635.59	422	1.51		
Resources	Scenario	610.56	1	610.56	478.05***	.53
	Scenario x Target	16.82	1	16.82	13.17*	.03
	Error	537.60	422	1.23		
Consent Given	Scenario	1484.79	1	1484.79	1018.92***	.71
	Scenario x Target	.88	1	.88	.60	.001
	Error	613.49	422	1.46		
Was Rape	Scenario	1590.63	1	1590.63	1047.77***	.71
	Scenario x Target	2.54	1	2.54	1.68	.004
	Error	639.12	422	1.52		

Note. †$p<0.1$ *$p<.05$ **$p<.01$ ***$p<.001$

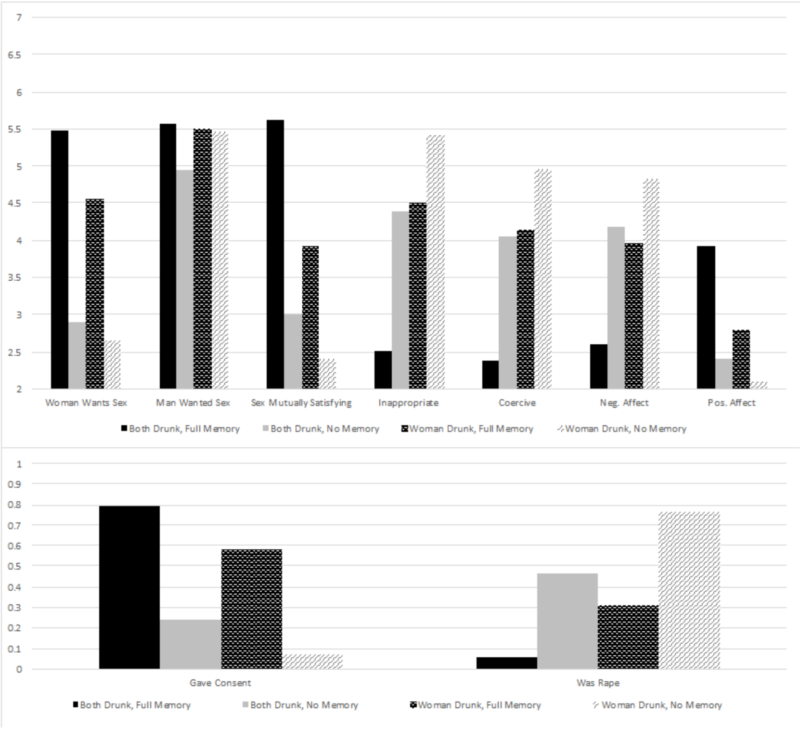

Figure S1. Scenario evaluations collapsing across perspective-taking condition for Study 1a. Significant contrasts are highlighted in Table 2. Higher scores indicate higher agreement.

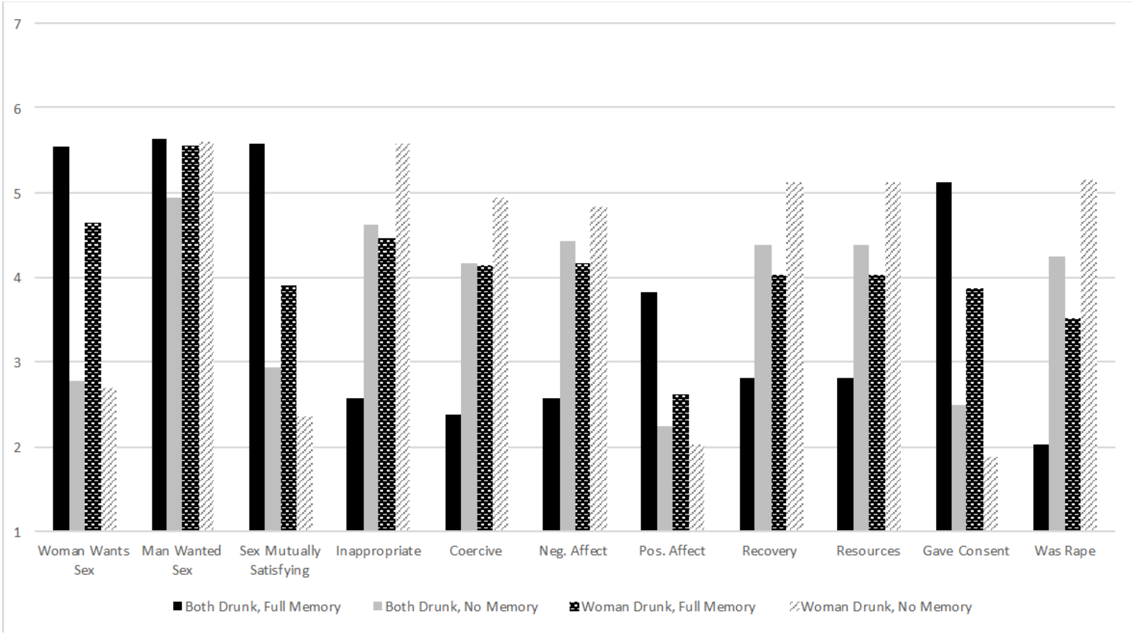

Figure S2. Scenario evaluations collapsing across perspective-taking condition for Study 1b. Significant contrasts are highlighted in Table 2. Higher scores indicate higher agreement.

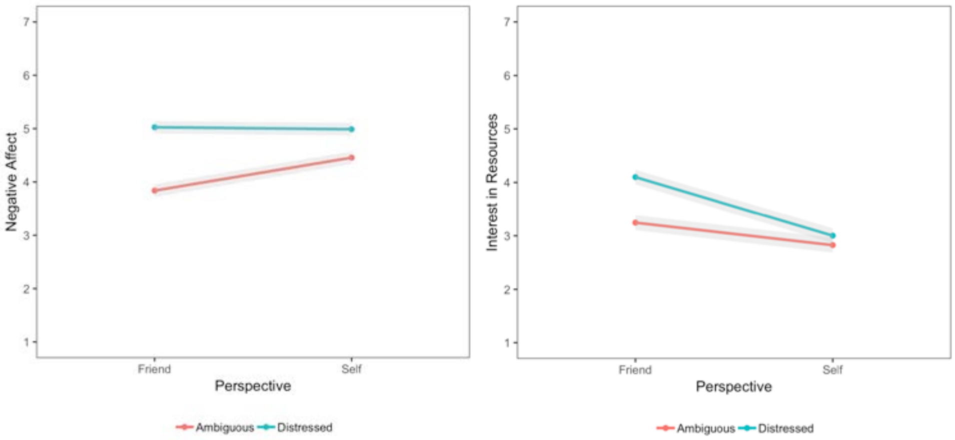

Figure S3. Study 2 two-way perspective-taking by distress condition interaction predicting negative affect and interest in resources.

SCENARIO INSTRUCTIONS & WRITING PROMPTS

Scenarios Studies 1a & 1b

Instructions Imagine Self Condition:

In the next part of the study you will be asked to imagine several scenarios resulting from a night out. Please immerse yourself in the details of each scenario and think about how you would think or feel in this situation. Please imagine that **YOU** are describing the events to a friend the next day.

Instructions Imagine Friend Condition:

In the next part of the study you will be asked to imagine several scenarios resulting from a night out. Please immerse yourself in the details of each scenario and think about how you would think or feel in this situation. Please imagine that **YOUR FRIEND** is describing the events to you the next day.

Scenario 1 (Both Intoxicated, Full Memory)

"Last night I went to a club with some friends from uni. It was so much fun. The music was really great and I started dancing with this hot guy called John. We were both so drunk! I completely lost count of how many drinks we both had. But he was really nice. It got a bit later and we decided to go back to his for another drink. We started kissing in his kitchen, and the next thing I knew, we were going hot and heavy in his bed. I hope we didn't wake his neighbours."

Scenario 2 (Both Intoxicated, No Memory)

"I decided to go out to the pub with some mates from work. While I was waiting at the bar for another drink, this guy Tom started chatting me up. Before I knew it, we were both so drunk-I'm afraid to look at how much I spent on drink! I must have agreed to go back to his because I woke up in his bed the next morning. It's been a while since I've had sex with someone. At least he was hot I guess."

Scenario 3 (Woman Intoxicated, Full Memory)

"I went for dinner with some friends after work. We ended up bumping into my friend's co-workers. One of them, Sean, was a right hotty. I was so excited that he was chatting me up that I totally lost count of how much I was drinking and got really, really drunk. Sean invited me back to his place, and we were all over each other. I haven't had sex in a long time. It was kind of awkward this morning because I realised Sean only had 1 or 2 drinks all night."

Scenario 4 (Woman Intoxicated, No Memory)

"I was at a mate's party and it was lit! I was having a really great time. At one point this really fit guy, Dan, started talking to me. I was SO drunk I don't even remember what we were talking about. Hopefully I didn't make a total fool of myself because Dan only had 1 or 2 drinks. I couldn't believe it when I woke up in his bed the next morning and realised we had sex the night before—I don't even remember leaving the party."

Scenario Study 2

"I decided to go out to the pub with some mates from work. While I was waiting at the bar for another drink, this guy Tom started chatting me up. Before I knew it, we were both so drunk-I'm afraid to look at how much I spent on drink! I must have agreed to go back to his because I woke up in his bed the next morning. It's been a while since I've had sex with someone. ***[I can't stop thinking about what we might have done. It makes me a bit sick to my stomach.]*** At least he was hot I guess."

Writing Prompt Study 3

First-person Perspective:
In this study, we are interested in the different types of sexual experiences people have had in their lives. In the next part of the study we would like you to write about the **worst** sexual experience you have ever had with a romantic partner. Please try to immerse yourself in the details of the experience. **When writing about this experience, please refer to yourself in the first person (e.g., "I did this; I felt this way").**

Distanced Perspective:
In this study, we are interested in the different types of sexual experiences people have had in their lives. In the next part of the study we would like you to write about the **worst** sexual experience you have ever had with a romantic partner. Please try to immerse yourself in the details of the experience. **When writing about this experience, please refer to yourself in the third person (e.g., "Mary did this; she felt this way").** In order to preserve confidentiality, you may use the names "Mary and James" when referring to yourself and your partner.

SCENARIO EVALUATIONS STUDIES 1a/1b & 2

1. **How much do you agree or disagree with each of the following statements regarding this scenario:**

   1=completely disagree, 7=completely agree

- You [your friend] really wanted to have sex with John.
- John really wanted to have sex with you [your friend].
- This encounter was mutually satisfying for both you [your friend] and John.
- Some of John's actions and behaviours were inappropriate.
- John used sexually coercive tactics to get you [your friend] to have sex with him.

2. **How would you [your friend] feel in in this scenario?**

   1=not at all, 7=extremely

- Nervous
- Angry
- Sad
- Stressed
- Guilty
- Frustrated
- Ashamed
- Happy
- Proud
- Enthusiastic
- Inspired
- Strong
- Active
- Interested

3. **Did you [your friend] give consent in this scenario?**

   1= Yes, 0 = No *[Study 1]*

   1= Definitely did not give consent, 7= Definitely gave consent *[Studies 2-3]*

4. **Did this scenario describe a rape?**

   1= Yes, 0 = No *[Study 1]*

   1= Definitely did not describe a rape, 7= Definitely described a rape *[Studies 2-3]*

The following questions were add in Studies 2&3:

5. **How long will it take you [your friend] to recover from or forget this experience?**

   1= Will recover/forget very quickly, 7=Will take a very long time to recover/forget

6. **Thinking back to the scenario, how likely are you to do each of the following? // Thinking back to the scenario, how likely are you to encourage your friend to do each of the following?**

   1=not at all likely, 7=extremely likely

- Find an online forum dedicated to woman's sexual experiences.
- Join an online community for women who have had bad sexual experiences.
- Read a leaflet about non-consensual sex and the resources available.
- Visit the website of a local sex crisis advocacy group.

- Look up the contact information for a local sex crisis centre.
- Speak with a counsellor about this sexual experience.
- File a police report about this sexual experience.

SCENARIO EVALUATIONS STUDY 3

1. **Next, we would like to ask you some questions about the experience you described. As a reminder, here is what you wrote […]:**

1=not at all, 7=extremely

- How much did you want to have sex with your partner?
- How much did your partner want to have sex with you?
- How satisfying was this experience for both of you?
- How inappropriate were your partner's actions and behaviours?
- How coercive was your partner during this experience?

2. **How did you feel during this experience?**

1=not at all, 7=extremely

- Nervous
- Angry
- Sad
- Stressed
- Guilty
- Frustrated
- Ashamed
- Happy
- Proud
- Enthusiastic
- Inspired
- Strong
- Active
- Interested

3. **Did you give consent in this scenario?**

1= Definitely did not give consent, 7= Definitely gave consent

4. **Do you think this experience could be described as a sexual assault?**

1= Definitely can not be described as a sexual assault,
7= Definitely can be described as a sexual assault

5. **How long will it take to recover from or forget this experience?**

1= Will recover/forget very quickly, 7=Will take a very long time to recover/forget

6. **Thinking back to the scenario, how interested would you be to learn more about the following services in your community?**

1=not at all interested, 7=extremely interested

- Find an online forum dedicated to woman's sexual experiences.

- Join an online community for women who have had bad sexual experiences.
- Read a leaflet about non-consensual sex and the resources available.
- Visit the website of a local sex crisis advocacy group.
- Look up the contact information for a local sex crisis centre.
- Speak with a counsellor about this sexual experience.
- File a police report about this sexual experience.

MEASURE OF SPONTANEOUS SELF-DISTANCING USED IN STUDIES 1-3.

Thinking back to the scenario(s) you just read, please indicate the extent to which you saw the events replay through your own eyes versus watched the event unfold as an observer, as you immersed yourself in the details of the experience.

1=Predominantly experienced through my own eyes,
7= Predominantly experienced as an observer
